# The range of peripapillary retinal nerve fibre layer and optic disc parameters in children aged up to but not including 18 years of age, as measured by optical coherence tomography: protocol for a systematic review

**DOI:** 10.1186/s13643-016-0247-z

**Published:** 2016-04-30

**Authors:** Alexandra L. Creavin, Cathy Williams, Kate Tilling, Nicholas Timpson, Julian P. T. Higgins

**Affiliations:** MRC Integrative Epidemiology Unit and Centre for Child and Adolescent Health, School of Social and Community Medicine, University of Bristol, Oakfield House, Oakfield Grove, Clifton, Bristol, BS8 2BN UK; Centre for Child and Adolescent Health, School of Social and Community Medicine, University of Bristol, Bristol, UK; MRC Integrative Epidemiology Unit, School of Social and Community Medicine, University of Bristol, Bristol, UK; School of Social and Community Medicine, University of Bristol, Bristol, UK

**Keywords:** Optic nerve, Retinal nerve fibre layer/retinal nerve fiber layer, Paediatrics / paediatrics, Ophthalmology, Normal range, Population, Optical coherence tomography (OCT)

## Abstract

**Background:**

The parameters of the optic disc and peripapillary retinal nerve fibre layer (pRNFL) in children may vary with disease processes that contribute to visual impairment and blindness and so could be useful as an objective measure in at-risk children. There is no standardised reference for the normal parameters of the optic disc and pRNFL in children; however, there are a large number of small individual studies that have been undertaken to look at these measures.

**Methods:**

A systematic review of current literature on the range of pRNFL and optic disc parameters in children aged less than 18 years will be performed. Studies will be considered for review if they report numerical data on optic disc and pRNFL parameters, measured using optical coherence tomography. Outcome measures will include mean pRNFL thickness and cup-disc ratio. The bibliographic databases Medline, CINAHL, EMBASE, Scopus and Web of Science will be systematically searched from 1991. Screening of search results will be conducted by two authors working independently, as will extraction of primary and secondary outcome data. Ten per cent of all other data extraction will be checked by a second author. Results will be compiled and presented in evidence tables. Where possible and appropriate, study-specific estimates will be combined to obtain an overall summary estimate of pRNFL thickness and cup-disc ratio across studies and results will be presented by age of population. Subgroup analyses will be undertaken for children of different ethnicities.

**Discussion:**

This review aims to provide an overview of the parameters of the optic disc and pRNFL in children of different ages in order to identify gaps in knowledge and to improve understanding of what might be considered within/outside the range of normality. The findings will be presented in peer-reviewed journals and will be presented at conferences.

**Systematic review registration:**

PROSPERO CRD42016033068

**Electronic supplementary material:**

The online version of this article (doi:10.1186/s13643-016-0247-z) contains supplementary material, which is available to authorized users.

## Background

### Rationale

In the United Kingdom (UK), around 1 in 500 children is visually impaired or blind [1]. Children can find it hard to understand or communicate a sight problem to others, particularly when they have other neurodevelopmental problems [2]. Objective measures of visual function can be difficult to assess with great accuracy and reliability in young children and frequently only identify gross impairment. Many causes of poor vision in children are thought to alter the appearance of the optic disc and the thickness of the peripapillary retinal nerve fibre layer (pRNFL) [3, 4]; however, optic disc and pRNFL morphology are poorly characterised in children, as was highlighted in a review in 2012 [5, 6].

### Objectives

The objective of this study is to identify what is currently known about the normal parameters of the paediatric optic disc and pRNFL, as measured by optical coherence tomography.

## Methods

### Eligibility criteria

#### Study characteristics

##### Population

Children who are aged up to but not including 18 years at the time of assessment and who do not have a known diagnosis affecting the eye or brain. Studies will be excluded if the results pertain only to a group of children with a specific exposure or pathology, e.g. children with cerebral tumours, children born prematurely, or children who have experienced facial trauma. Studies involving adult participants will be included if it is possible to extract data that is pertaining only to children.

##### Outcome

1. Peripapillary retinal nerve fibre layer (pRNFL) parameters (mean thickness, thickness of quadrants), measured using optical coherence tomography (OCT).
2. Optic disc parameters (disc area, vertical and horizontal disc height, cup size and resultant cup-disc ratio and neuro-retinal rim area, obliquity) quantified by OCT.

Studies will be excluded if the measurements are not taken using OCT.

##### Types of study

Cross-sectional, cohort studies and control groups of case-control studies will be included. In the case of randomised controlled trials, it will be possible to include control-arm information and to include intervention group information, where the intervention would not affect the parameters of interest.

If sufficient population-based or prospective studies are available, these will be used in isolation. If it is necessary to include convenience or retrospective samples, these will be assessed for selection bias. Reviews, case reports and case series will not be included.

#### Report characteristics

##### Years considered

Databases will be searched from 1st January 1990 onwards, as OCT was not developed until 1990 [7].

### Language

There will be no limitations on language as long as the title can be searched using English language keywords.

### Publication status

Literature that is published online or in print will be included.

### Other restrictions

Articles will only be included in the analysis where there are numerical measures of optic disc or pRNFL parameters.

### Information sources

#### Electronic databases

The electronic databases used were Medline (via Ovid), CINAHL, EMBASE (via Ovid), Scopus and Web of Science.

### Other

References lists will be searched (see the “Search strategy” section below). The authors will also contact experts in the field for their unpublished data.

### Search strategy

#### Example

(optic nerve? OR neuro?retinal rim OR nerve fiber layer? OR nerve fibre layer? OR RNFL? OR stratum opticum OR retinal nerve fiber? OR retinal nerve fibre? OR optic disc? OR optic disk? OR optic cup? OR cup-disc? OR cup-disk? OR nerve head? OR cupping) AND (spectral domain OR fourier domain OR optical coherence OR optical coherent?) AND (paediatric? OR pediatric? OR highschool? OR high school? OR secondary school? OR student? OR youth? OR young OR teen? OR prepubescent OR pre-pubescent OR pubescent OR puberty? OR preadolescent OR pre-adolescent OR adolesc? OR minors? OR juvenile? OR elementary school? OR primary school? OR schoolchild? OR schoolage? OR school-age? OR kids OR child? OR preschool? OR pre-school? OR nursery school? OR toddler? OR infant? OR babies OR newborn? OR neonat? OR girls OR boys) NOT (animals NOT humans[mesh terms])

Paediatric keywords were determined by compiling a combination of the Cochrane Child Health Field [8] with a University of Bristol paediatric search strategy which has been developed over a number of years.

### Study records

#### Data management

Records will be managed using EndNote.

### Selection process

#### Screening

Studies identified by the search strategy will be screened in EndNote. Duplicates will be removed, and titles and abstracts will be screened by two members of the study team working independently. Disagreements will be resolved by discussion between them, with the option of further discussion with a third team member as required.

#### Eligibility

Full text articles will be assessed for eligibility by two members of the study team working independently. Any disagreement between the two authors will be resolved by discussion between them, with the option of further discussion with a third team member as required.

Of the full text papers selected for inclusion, the authors will search the reference lists of a random sample of five papers to screen for further work for inclusion.

### Data collection process

Data will be extracted by two members of the study team working independently. Data extraction forms will be piloted on a sample of five papers. A proposed list of data to be extracted is given (Additional file 1).

Where required and feasible, the lead author will communicate with investigators of published studies in order to obtain or confirm data.

### Outcomes and prioritisation

#### Main outcomes

1. pRNFL: mean pRNFL thickness
2. Optic disc: cup-disc ratio

These are the outcomes most likely to be recorded by a large number of studies. They are also used clinically on a regular basis and so are directly applicable to the clinical practice. There is likely to be limited heterogeneity in how these variables are expressed. The outcomes will be summarized using means and standard deviations where possible and appropriate. If distributions of the outcome measurements are skewed, they may be reported using other statistics such as medians or geometric means, with interquartile ranges or ranges. Distributions will be summarized on the natural scale of the outcome, taking into account the possibility of skew.

### Additional outcomes

1. pRNFL: segmental pRNFL thickness (as quadrants or clock-hours depending on data available).
2. Optic disc: optic disc area, optic disc height, optic cup size; neuroretinal rim area; obliquity.
3. Global and central field macular thickness.

These variables provide a more detailed picture of the optic disc and pRNFL. They are not likely to be reported as frequently as the main outcomes and are less immediately applicable in a clinical setting.

### Quality of individual studies

Data relating to the methodological quality of individual studies will be extracted as part of the data extraction process (see Additional file 2), including details of how individuals were selected into the study, the basis for exclusion from the study, scan quality and the use of published acquisition protocol such as the OSCAR-IB [9]. The findings of this assessment will be used to inform a sensitivity analysis of high quality studies, i.e. studies that satisfy at least four of the five quality criteria.

### Data synthesis

Tables will be compiled giving descriptive information for each included study. These will describe the population examined, the examination protocol used, including machine make and model and the baseline characteristics of participants.

A descriptive and graphical presentation of the individual study estimates of main outcomes will be given to include means and standard deviations with different makes and model of machine highlighted. Measurements made using time domain and spectral OCT devices and different makes or models of OCT machine will be compared using sensitivity analyses. If sufficient studies have used the same brand and model of OCT machine in children of a comparable age, study-specific estimates will be meta-analysed to obtain an overall summary estimate of pRNFL across studies, by age of population. Subgroup analyses will be undertaken for children of different ethnicities. pRNFL quadrant data will be compared and likewise for clock-hour sectors, unless it is possible to reliably assign the clock hours to a quadrant.

### Report of the review

Report of the review will follow the PRISMA guidelines. A Prisma-P checklist is included with this manuscript along with a list of data collection points (see Additional file 3).

### Ethics approval and consent to participate

Not applicable.

### Consent for publication

Not applicable.

## Additional files

Additional file 1: Proposed list of data for extraction from full text articles. (DOCX 15 kb) Additional file 2: Proposed list of quality criteria. (DOCX 17 kb) Additional file 3: Prisma-P checklist. (DOCX 36 kb)

## Abbreviations

OCT: optical coherence tomography
pRNFL: peripapillary retinal nerve fibre layer
