# Supplementary material for: The range of peripapillary retinal nerve fibre layer and optic disc parameters in children aged up to but not including 18 years of age, as measured by optical coherence tomography: protocol for a systematic review
Source: Syst Rev. 2016 Apr 30;5:71. doi: 10.1186/s13643-016-0247-z (PMC4853851; doi:10.1186/s13643-016-0247-z)
Supplement: Additional file 3: — Prisma-P checklist. (DOCX 36 kb) [file 13643_2016_247_MOESM3_ESM.docx]

Quality criteria

1. Population-based
2. Prospective
3. Known ophthalmic disease excluded / eye examination done and gross abnormality excluded
4. Known cerebral/neurodevelopmental disorder excluded
5. OCT measurement involved use of a published acquisition protocol, (in particular signal strength was 6 or more, where the machine had this capacity).
